# Supplementary material for: Modeling and simulating the multi-generation product sales, production and inventory system within the context of quality upgrades
Source: PLoS One. 2024 Apr 5;19(4):e0299944. doi: 10.1371/journal.pone.0299944 (PMC10997069; doi:10.1371/journal.pone.0299944)
Supplement: Attachment — Submitted filename: Comments.docx [file pone.0299944.s002.docx]

Based on the Norton-Bass model, this paper establishes a comprehensive multi-generation product diffusion sales and supply system model focusing on quality upgrading, and simulates and solves the system under the decentralized and centralized decision-making modes respectively by using the system dynamics theory, and further compares and analyzes the two decision-making modes. This paper reveals the internal mechanism of product renewal in enterprises from the dimensions of production and inventory, quantifies the important impact of quality upgrading on brand influence, and clarifies the relationship between quality upgrading and product brand influence. This paper finds that a centralized decision optimization model improves profitability. In order to maximize profits, both order backlogs and inventory shortages contribute to the overall profitability of the firm.

In conclusion, this paper makes a valuable contribution to the field by investigating the internal mechanism of multigenerational product renewal and its impact on the overall decision-making of a firm by modeling a comprehensive multigenerational product diffusion sales and supply system in the context of product quality upgrading, taking into account both production and inventory aspects. The methodology employed, including the use of system dynamics simulations and the Stackelberg model, enhances the reliability of the findings. The conclusions provide insight into the complex relationship between multigenerational product quality upgrading and overall firm decision-making, providing practical implications for revealing the internal mechanisms of product renewal in firms.

However, this manuscript could be improved in the following ways:

1. In order to strengthen your literature review and theoretical implications, you may want to include more recent and relevant references published in recent months (years), which currently appear to be farther back in time for this paper.

2. Try to illustrate your findings in the literature review section by comparing it with the existing literature. The literature review section should be presented logically and try to explain the more novel ideas in your study compared to the existing literature. Provide a table of author contributions in this section to highlight the unique novelty of this paper.

3. The profit function in the model section is confusing, try to explain which are costs, which are revenues and which are profits. Equations (7-9) confuse me as well, if the first derivative in equation (7) should be dS_1(t)/dt as stated in the text?

4. The authors should add a "real" discussion section with extensive comments on the results obtained. I would like to know how the results obtained in this paper are similar to or different from other studies, which are currently unclear.

5. Please use the correct styles for all formula inputs and tables in the article.
